# Biological and Clinical Characteristics of Proximal Colon Cancer: Far from Its Anatomical Subsite

**DOI:** 10.7150/ijms.97574

**Published:** 2024-07-09

**Authors:** Qing Yang, Ruize Qu, Siyi Lu, Yi Zhang, Zhipeng Zhang, Wei Fu

**Affiliations:** 1 Department of General Surgery, Peking University Third Hospital, Beijing China.; 2 Cancer Center, Peking University Third Hospital, Beijing China.

**Keywords:** Colorectal cancer, Proximal colon cancer, Right-sided colon cancer, Tumor biology, Gut microbiota

## Abstract

Colorectal cancer is a heterogeneous disease which can be divided into proximal colon cancer, distal colon cancer and rectal cancer according to the anatomical location of the tumor. Each anatomical location of colorectal cancer exhibits distinct characteristics in terms of incidence, clinical manifestations, molecular phenotypes, treatment, and prognosis. Notably, proximal colon cancer differs significantly from cancers of other anatomical subsites. An increasing number of studies have highlighted the presence of unique tumor biological characteristics in proximal colon cancer. Gaining a deeper understanding of these characteristics will facilitate accurate diagnosis and treatment approaches.

## 1. Introduction

Colorectal cancer (CRC) is a disease characterized by its heterogeneity and distinct anatomical, pathophysiological, and clinical attributes. In the United States, CRC ranks third in both incidence (35.9/100,000 person-years) and mortality (13.1/100,000 person-years), according to the American Cancer Society^1^. Although regional disparities exist, the overall morbidity and mortality rates are higher among men than women.

According to the anatomical subsite, CRC is categorized into proximal colon cancer (also known as right-sided colon cancer, from the ileocecum to the proximal 2/3 of the transverse colon), distal colon cancer (also known as left-sided colon cancer, from the distal 1/3 of the transverse colon to the sigmoid colon), and rectal cancer (RC). Numerous studies conducted over the past three decades consistently demonstrated substantial variations in the incidence, clinical manifestations, pathological classifications, and prognoses of CRC across different anatomical locations^2^,^3^. An examination of these distinct manifestations from an embryological standpoint elucidates that the proximal colon arises from the midgut during embryonic development, while the distal colon originates from the hindgut^4^. Consequently, this divergence in origin gives rise to dissimilar histological composition and physiological functionality^5^. As a consequence, tumors develop in a distinct trajectory with distinctive molecular characteristics^6^,^7^. Hence, scholars have posited that the proximal colon and the distal colon are regarded as “two organs derived from a common intestinal canal”, thereby advocating for more precise therapeutic interventions contingent upon the anatomical site of the tumor.

In relation to clinical presentations, individuals diagnosed with proximal colon cancer demonstrate a greater prevalence of females in comparison to those diagnosed with distal colon cancer or RC. Moreover, they manifest a higher incidence of symptoms, including abdominal pain and palpable masses, particularly in cases where the tumor diameter is larger^3^. Furthermore, it has been observed that patients diagnosed with proximal colon cancer tend to have a more unfavorable prognosis compared to those with distal colon cancer or RC. This finding has been consistently reported across various studies. For instance, a study conducted on a U.S. patient population^3^ revealed that proximal colon cancer exhibited lower 5-year overall survival (OS) and 5-year disease-free survival (DFS) rates compared to distal colon cancer and RC, specifically in AJCC stage I and III cases. Similarly, a study conducted in Chinese patient population^8^ demonstrated that proximal colon cancer patients had a significantly lower 5-year cancer-specific survival (CSS) rate compared to distal colon cancer (68.1% vs. 70.9%, p < 0.001). The pathological characteristics of proximal colon cancer distinguish it from both distal colon cancer and RC, as they frequently exhibit poorly differentiated tumors^9^, mucinous adenocarcinoma^10^, and signet-ring cell cancers^11^. Additionally, proximal colon cancer displays a distinctive molecular pathology, often characterized by a higher prevalence of BRAF mutations and microsatellite instability-high (MSI-H)^12^. Consensus molecular subtype (CMS) divided CRC into 4 different types CMS1-4^13^. The proportion of CMS1 and CMS3 was higher in proximal colon cancer than in distal colon cancer and RC^14^.

The management approach for proximal colon cancer exhibits variations when compared to both distal colon cancer and RC. Surgical intervention continues to be the primary therapeutic modality for proximal colon cancer patients. According to the NCCN guidelines^15^, the molecularly-targeted therapies cetuximab and panitumumab demonstrate a diminished prognostic advantage for proximal colon cancer patients compared to those with distal colon cancer in the context of CRC. Consequently, these therapies are presently recommended exclusively as first-line treatments for patients with distal colon cancer. At present, a dearth of systematic reviews pertaining to the distinctive attributes of proximal colon cancer persists. This paper aims to consolidate the epidemiological, clinical, tumor biological, and therapeutic characteristics of proximal colon cancer, thereby offering a valuable point of reference for the advancement of proximal colon cancer-related treatment and research.

## 3. Epidemiology

The composition ratio of CRC exhibits regional and ethnic variations across different anatomical sites. According to data from the American Cancer Society, proximal colon cancer constituted 39%^1^ of all CRC cases in the United States. Similarly, a study encompassing 10 European countries reported a ratio of 29.8%^16^. Conversely, a Chinese study proposed a ratio of 20%^17^, while a previous study conducted at our center yielded a comparable proximal colon cancer ratio of 18.6%^18^. Japanese and Korean studies concluded ratios of 14.0%^19^ and 23.8%^20^, respectively. As seen in the above studies, the prevalence of CRC varies among different sites in different national populations, with relatively consistent statistics among Asian countries, where the proximal colon is the less common site of CRC; whereas in Europe and the United States, the proximal colon is the more common site, and the proximal colon cancer constitutes the highest ratio of CRC among all anatomical sites even in American patients. The difference ratio of proximal colon cancer in CRC among countries may can be explained by metabolic syndrome (MetS). A population-based cohort study in Norway demonstrated that MetS was associated with proximal colon cancer rather than distal colon cancer or RC^21^. A cohort-study in China also found similar conclusion^22^. Prevalence of MetS in the United Status among adults is about 34.7%^23^, the number in the Unit Kingdom is 31.1%^24^, while in China the number is about 15.5%^25^. Therefore, MetS contributing to regional difference proximal colon cancer incidence. And the advocation of healthy lifestyles play a potential role of primary prevention in proximal colon cancer. However, regardless of the region, proximal colon cancer patients have a higher proportion of females compared to distal colon cancer and RC, ranging from 45.9%-64.4%^1^,^3^,^8^,^16^ and have the highest mean age at diagnosis and the latest detection time^3^.

A comprehensive analysis of three decades of data from the United States revealed a decreasing overall incidence rate of CRC. However, the decline in proximal colon cancer was notably less pronounced compared to distal colon cancer and RC, indicating a discernible upward trend in the proportion of proximal colon cancer within the broader context of CRC^26^. This may be due to improvements in diagnostic and treatment techniques, as well as the widespread use of colonoscopy, which has increased screening and removal of adenomatous polyps in the distal colon, which has resulted in better prevention of distal colon cancer and a reduction in the incidence of distal colon cancer. However, colonoscopy was not as effective in preventing proximal colon cancer as it was in preventing distal colon cancer due to suboptimal bowel preparation of the proximal colon, incomplete colonoscopy, and a higher incidence of serrated polyps in the proximal colon^27^. Overall, the proportion of proximal colon cancer in CRC is on the rise, and the anatomical sites where CRC occurs have a “rightward shift” trend^28^.

## 3. Clinical Features

Patients with early-stage CRC usually have no clinical presentations, and diagnosis of CRC during the asymptomatic period is often a screening finding. Most patients with CRC seek medical treatment after the onset of symptoms Clinical symptoms are often caused by the tumor growing into the intestinal lumen and then invading the intestinal wall even the surrounding adjacent tissue structures. Therefore, the occurrence of clinical symptoms often indicates that the tumor has progressed and is no longer in the early stages.

Among typical clinical signs or symptoms of CRC^29^, the most common clinical symptoms of proximal colon cancer are abdominal mass and anemia; while the most common clinical symptoms of distal colon cancer are hemorrhage and obstruction. It is currently believed that the reason for these differences is that the proximal colon has a larger intestinal lumen and higher fecal water content, making it less likely to be obstructed, while the distal colon has a relatively smaller intestinal lumen, lower fecal water content, and more common symptoms of obstruction.

In addition, different tumor locations have different prognosis^30^. Weiss *et al.*^31^ analyzed stage I to III colon cancer patients in the Surveillance, Epidemiology, and End Results-Medicare Data (SEER). The results showed that both patient characteristics and tumor characteristics are factors affecting patient survival, among which tumor stage is an independent predictive factor. Adjusted Cox regression showed no significant difference in 5-year mortality between proximal and distal colon cancers for all stages combined or for stage I cancers. Stage II proximal colon cancer had lower 5-year mortality than distal colon cancer, and stage III proximal colon cancer had higher 5-year mortality. Compared with distal colon cancer and RC, patients with proximal colon cancer have lower 5-year OS and 5-year DFS. This may be due to the fact that microsatellite instability (MSI) is mainly seen in proximal colon cancer (while less than 5% of distal colon cancers have MSI), and MSI positivity is associated with tumors at earlier stages.

Jernvall *et al.*^32^ conducted a statistical analysis and determined that the prevalence of MSI-positive patients in stage II proximal colon cancer ranged from 20% to 25%, while in stage III proximal colon cancer patients, it was less than 15%. Furthermore, the proportion of MSI-positive patients in stage IV colon cancer was found to be even lower. In a separate study, Yahagi *et al.*^33^ found that patients with proximal colon cancer generally exhibited a poorer prognosis, particularly those in AJCC stage III/IV. This study also revealed that this disparity is more pronounced in Western nations but exhibits inconsistency in Eastern countries, potentially attributed to factors such as race^20^,^34^, lifestyle^35^-^37^, healthcare policies, and other pertinent determinants.

## 4. Tumor Biological Characteristics

The biological behavior of proximal colon cancer differs from that of distal colon cancer. In terms of tumor morphological characteristics, proximal colon cancer tumors typically exhibit a larger diameter, a higher proportion of pathological stage II, poorer differentiation compared to distal colon cancer and RC, and an increased incidence of lymphovascular invasion^3^. From the perspective of specific histological classification, mucinous carcinoma, signet ring cell carcinoma and medullary carcinoma in proximal colon cancer is more common than that in distal colon cancer or RC^3^,^38^-^40^.

This observation may be attributed to the unique embryonic origin, which can influence cells by differential gene expression leading to divergent patterns of differentiation. This leads to distinct histological structures and physiological functions in the distal and proximal colons^5^. Consequently, these variations significantly impact tumor occurrence and progression, resulting in diverse symptoms, molecular characterization, and biological behavior^6^,^7^.

In addition, the development of CRC is intricately influenced by the interplay between genetic factors and the tumor micro-environment (TME). It is worth noting that variations in TME characteristics across different anatomical locations of CRC may also contribute to the distinctive tumor biological features observed in proximal colon cancer.

### 4.1 Molecular Biology

There are two major distinct precursor lesion pathways of sporadic CRC at the molecular level. One is the conventional adenoma to carcinoma pathway^41^, also referred to as the chromosomal instability sequence, which accounts for 70-90% of CRC. The other one is the serrated neoplasia pathway^42^, contributing to 10-20% of CRC. The serrated neoplasia pathway can be categorized into two types: traditional serrated adenomas (TSA) and sessile serrated polyps (SSP), among them, 80% of SSP caused proximal colon cancer. Initiation of the serrated neoplasia pathway typically involves genetic mutations in the BRAF or KRAS genes, followed by tumor suppressor gene methylation known as CpG island methylator phenotype (CIMP). These processes can result in microsatellite-stable (MSS) and MSI tumors depending on the epigenetic silencing of specific genes during lesion progression.

#### 4.1.1 MMR, RAS, BRAF

There are individual differences in the expression of proto-oncogenes and anti-oncogenes between proximal colon cancer and distal colon cancer. Prevalent mutations in proximal colon cancer^43^-^45^ involve the following genes: PIK3CA, FBXW7, SMAD4, TGFBR2, BRAF, CTNNB1, PTEN. Recent studies^46^ also support that there are differences in the genetic architecture of colon cancers across distinct anatomical locations, manifested in multiple gene loci, which implies that the molecular mechanisms involved in the development of tumors in different locations need to be discussed separately, however, a definitive conclusion is yet to be reached. In conjunction with the aforementioned pathogenic mechanism in molecular biology, significant focus has been placed on MMR-related genes as well as RAS and BRAF as valuable molecular markers for clinical diagnosis and treatment of CRC.

##### 4.1.1.1 MMR

MMR is a mismatch repair protein, and when this protein is functionally defective, it is referred to as dMMR. Typically, genetical characteristics of dMMR tumors including the inactivation of genes MLH1, MSH2, MSH3, MSH6, and PMS2; epigenetic inactivation; and downregulation of microRNA. Overall, hypermethylation of the MLH1 promoter represents the primary mechanism underlying MSI-H in sporadic CRC, including BRAF-mutated CRC^47^,^48^. Hypermethylation of the MLH1 promoter is exclusively observed in SSP and is associated with specific polymorphisms in MLH1 (MLH1-93AA)^49^. In patients with restrictive CRC, a higher proportion of MSI-H occurs in proximal colon cancer compared to distal colon cancer; furthermore, patients with MSI-H tumors exhibit a more favorable survival rate. This finding may elucidate the better prognosis of patients with stage II proximal colon cancer relative to those with distal colon cancer. It should be noted that the prevalence of MSI-H is lower among individuals diagnosed with metastatic colorectal cancer (mCRC), and the impact on prognosis remains uncertain.

##### 4.1.1.2 RAS

RAS can be categorized into three groups: HRAS, KRAS and NRAS. Although all three oncogenes have the ability to transform normal cells when mutated, KRAS mutations are the most prevalent in human CRC^50^,^51^. Notably, RAS gene mutations are more frequently observed in proximal colon cancer^52^. In CRC, RAS gene mutations are significantly associated with a lack of response to drugs targeting the epidermal growth factor receptor (EGFR) such as cetuximab^53^. In other words, only patients with wild-type RAS genes can benefit from anti-EGFR therapy^54^. The proportion of such wild-type patients is lower in proximal colon cancer compared to distal colon cancer and RC.

##### 4.1.1.3 BRAF

BRAF activating mutations (mostly occurring at codon 600, i.e. V600E): CRCs with BRAF^V600E^ mutations account for 8% to 12%^55^ of all diagnosed CRC cases, of which approximately 60% of primary tumors are located in the proximal colon. BRAF^V600E^ serve as a strong adverse prognostic indicator for early-stage and late-stage/recurrent non-MSI-H tumors due to its role in conferring resistance to anti-EGFR therapy. However, MSI-H tumors (where most BRAF mutations occur) do not share this poor prognosis despite harboring BRAF mutations; neoadjuvant chemotherapy still demonstrates better efficacy^47^,^56^,^57^.

Current large-scale clinical studies have established that mutation status of RAS and BRAF genes as well as MMR status are dominant prognosis or predictive biomarkers, and RAS, BRAF, and MMR status should be detected to obtain information on patient risk stratification and optimize treatment options^58^,^59^. These also become part of the routine pathological assessment for CRC. While BRAF mutations and MSI were more common in proximal colon cancer patients, this was only less than 5% in the distal colon cancer patient population. BRAF mutations and MSI are associated with poorly differentiated adenocarcinoma, mucinous carcinoma, and signet ring cell carcinoma. MSI is not only associated with a better prognosis after radical resection in stage II patients, but also with fewer liver metastasis. For example, in stage IV proximal colon cancer patients, liver metastases are significantly less common, while peritoneal metastasis is more common^60^.

#### 4.1.2 PIK3CA, SMAD4, CTNNB1, PTEN

In addition to the aforementioned genes, numerous biomarkers have been extensively investigated; however, their diagnostic and prognostic value remains unproven. This is primarily attributed to inconsistent assay methodologies, conflicting findings across different studies examining the same factor, as well as a predominance of small-scale studies lacking statistically robust and valid multivariate analysis. However, such genes or biomarkers still have important research value and are differentially expressed in proximal colon cancer. Here are a few categories for a brief introduction (Table 1).

##### 4.1.2.1 PIK3CA

PIK3CA is a common oncogene that encodes the p110α catalytic subunit of class I phosphatidylinositol-3-kinases (PI3Ks), namely PI3Kp110α, which is involved in cancer driving mechanisms mainly as an upstream signal for AKT. Cancers with high activation rates of PIK3CA mutations include breast cancer (>30%), endometrial cancer (>30%), bladder cancer (>20%), CRC (>17%), and head and neck squamous cell carcinoma (>15%)^61^. A study^62^ suggested that PIK3CA mutations were associated with older age, proximal colon cancer, and histological classification of mucinous carcinoma and KRAS mutation. Among the hotspot locations were exons 9 and 20 of PIK3CA. In the comprehensive cohort analysis, PIK3CA exon 9 and 20 mutations were overrepresented in proximal colon cancer, CIMP-low (CIMP-L), and KRAS-mutated cancers. Comparing PIK3CA exon mutations, exon 20 mutations were associated with MSI-H, CIMP-H and BRAF mutations, while exon 9 mutations were associated with KRAS mutations. A similar study including 757 CRC patients reached the same conclusion and also found that CRC with PIK3CA mutations were more likely to be associated with deficient expression of O-6-methylguanine-DNA methyltransferase (MGMT), which also leads to a significant decrease in the survival rate of patients with BRAF wild-type tumors^44^.

##### 4.1.2.2 SMAD4

SMAD4 is a tumor suppressor and a crucial component of the transforming growth factor-β (TGF-β) signaling pathway, playing significant roles in cell proliferation, differentiation, migration, apoptosis, and interaction with stromal inflammatory cells.

One prospective targeted sequencing study^63^ on 1134 CRC patients revealed that compared with distal colon cancer, MSS mCRC with the primary tumor located in the proximal colon is associated with worse survival rates, older age at diagnosis, increased mutations, and enrichment of oncogenic mutations in KRAS, BRAF, PIK3CA, AKT1, RNF43, and SMAD4, which also suggests differences in tumor metastasis mechanisms between proximal colon cancer and distal colon cancer.

Interestingly, in another study^64^ involving 108 patients comparing colorectal adenocarcinoma with mucinous component (AWMC) and classic colorectal adenocarcinoma (AC), the most frequently mutated genes identified in AWMC were KRAS (45.4%), TP53 (39.8%), APC (22.2%), PIK3CA (22.2%) and SMAD4 (10.2%). Furthermore, AWMC was found to be more prevalent in proximal colon cancer. The conclusion of this study provides an explanation for the higher frequency of SMAD4 mutations observed in proximal colon cancer from a histological classification perspective.

##### 4.1.2.3 CTNNB1

CTNNB1 encodes β-cantenin, which can drive carcinogenesis upon abnormal activation. When oncogenic mutations occur, the produced protein resists proteolytic degradation and activates the Wnt signaling pathway to promote tumorigenesis. A study^65^ that included 30 patients with sporadic CRC and 17 patients with hereditary non-polyposis colorectal cancer (HNPCC) revealed a higher prevalence of CTNNB1 mutations in the proximal colon, independent of MSI status. In another study^45^ comprising 1,876 patients with CRC, CTNNB1 mutations were predominantly observed in the proximal colon; however, further subdivision based on tumor location demonstrated that tumors located at the splenic flexure exhibited the highest frequency of CTNNB1 mutations.

##### 4.1.2.4 PTEN

The phosphatase and tensin homologue deleted on chromosome ten (PTEN), also known as MMAC1 and TEP1, is a classic tumor suppressor gene belonging to the protein tyrosine phosphatases (PTP) gene family. PTEN protein exerts its tumor-inhibiting effects by antagonizing the activity of phosphorylase enzymes such as tyrosine kinases. Deletion or mutation of the PTEN gene leads to tumorigenesis. In a study^62^ involving 1093 patients with stage I-IV colorectal cancer, PTEN mutations were detected in 5.8% of clinical samples. The results indicated that PTEN mutations are associated with proximal colon tumors, mucus histology, MSI-H, CIMP-high (CIMP-H), and BRAF mutation. Furthermore, another study^66^ examined PTEN and PIK3CA mutations in 186 adenocarcinomas and 16 adenomas from the EPIC Norfolk study using DNA sequencing and assessed changes in PTEN expression through immunohistochemistry. This study identified mutations in exons 7 and 8 of the PTEN gene in 2.2% of CRC cases, along with loss of PTEN expression in 34.9% of CRC cases; notably, loss of PTEN expression exhibited heterogeneity across different anatomical locations: proximal colon cancer was associated with advanced Dukes stage and lower differentiation level, while distal colon cancer showed an association with earlier Dukes stage. These findings suggest variations in molecular biology among different hemi-tumors and highlight the differential diagnostic value of PTEN across various anatomical locations within CRC.

#### 4.1.3 Consensus Molecular Subtypes

In 2015, CMS classification of CRC was proposed internationally^13^: CMS1 (MSI Immune, 14%), CMS2 (Canonical, 37%), CMS3 (Metabolic, 13%), CMS4 (Mesenchymal, 23%), and samples with mixed features (13%). Whereas CMS typing has shown variation according to the anatomical location of CRC, more studies have been conducted and reached a consensus that CMS type 1 and 3 are more prevalent in proximal colon cancer^12^,^14^,^67^,^68^. This finding aligns with the observation that proximal colon cancer exhibits a higher proportion of MSI, CIMP, KRAS mutations, and BRAF mutations. The differences in CMS typing are significant as they contribute to the understanding of distinct tumor biological behaviors between proximal colon cancer and distal colon cancer, offering novel insights and a comprehensive summary of the distinct gene mutation characteristics.

### 4.2 Tumor Micro-Environment of proximal colon cancer

#### 4.2.1 Gut Microbiota

In the human gut, colonies of over 10^14 microorganisms^69^ play crucial roles in maintaining a normal physiological environment, including energy metabolism, interaction with the intestinal barrier system, promotion of epithelial cell survival and most importantly protection against external or pathogenic bacterial invasion^70^. Dysbiosis in the gut has been linked to various diseases such as neurological disorders, gastrointestinal issues and metabolic conditions^71^. Changes in dietary habits or environmental factors can induce alterations in the gut microbiota^72^ which may lead to colorectal cancer through inflammation, DNA damage, or metabolites produced from microorganisms^73^,^74^.

**Figure 3:**
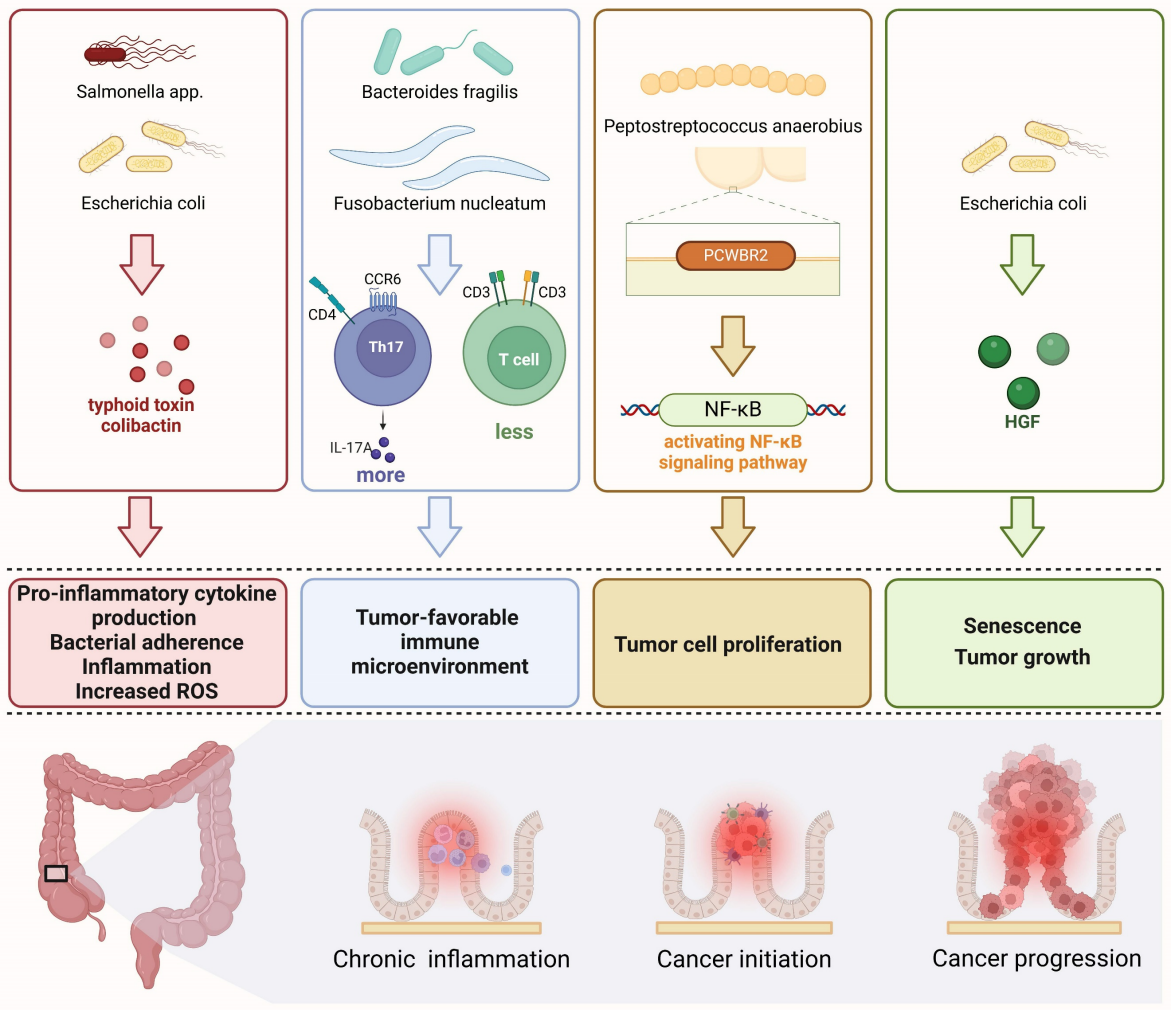
** Four key mechanisms of gut microbiome associated with the occurrence and progression of colorectal cancer** a. The inflammation pathway triggered by toxins secreted by the gut microbiome, such as Salmonella or E. coli, can lead to chronic inflammation and an increase in reactive oxygen species (ROS) within the mucosal lining, resulting in DNA damage. b. Tumor microenvironment (TME): Certain microorganisms like Fusobacterium nucleatum and Bacteroides fragilis have the ability to modulate the density of different types of T cells, creating a tumor microenvironment that promotes tumor initiation and progression. c. Bacterial components can promote tumor cell proliferation through the NF-κB pathway, for example, via putative cell wall binding repeat 2 (PCWBR2) surface protein produced by Peptostreptococcus anaerobius. d. Senescence and tumor growth can be facilitated by specific strains of E. coli that produce colistin-encoded enzymes responsible for hepatocyte growth factor (HGF) synthesis.

Gut microbiome influences the onset and progression of CRC by chronic inflammation^75^, tumor-favorable immune microenvironment^76^-^78^, promoting CRC tumor cells proliferation^79^, inducing senescence and promoting tumor growth^80^ (as shown in Fig. 3).

Regarding colon cancers at different sites, several relevant studies have demonstrated significant differences in the composition of intestinal mucosal microbiota^81^,^82^ and bacterial biofilm^83^. In terms of microbial species, Prevotella, Pyramido-bacterium, Selenomonas, and Peptostreptococcus were found to be more prevalent in proximal colon cancer^81^. Biofilm refers to a mixed bacteria mucin layer present on the surface of the epithelial lumen of the colon. A study by Dejea *et al.*^83^ revealed that invasive bacterial biofilms were detected in 89% of proximal colon cancer cases compared to only 12% in distal colon cancer cases; furthermore, most patients with biofilms on their tumors also exhibited similar invasive biofilms on normal colonic mucosa distant from the tumor site. The biofilms were associated with a significant decrease in epithelial E-cadherin, increased interleukin-6 (IL-6), activated Stat3 signaling pathway, enhanced cell proliferation, and elevated levels of N1, N12-diacetylspermine. This suggests a unique symbiotic relationship between bacterial biofilms and host cancers.^84^.

Additionally, relevant investigations have been conducted to classify the intestinal microbiota based on carcinogenesis in sporadic colon cancer and identify disparities between TSA and SSP mechanisms^85^, with proximal colon cancer accounting for 80% of SSP-related carcinogenesis^86^. A comprehensive review summarizing pertinent studies has established a distinct correlation between Fusobacterium spp. and proximal lesions characterized by higher histologic grading as well as serrated pathway lesions. Yu *et al.*^87^ found that Fusobacterium spp. was more prevalent in SSP than in adenomas, and more common in proximal than distal CRCs. While Ito *et al.*^88^ did not observe differences in Fusobacterium spp. between adenomas, TSA, or SSPs, their group did find that the proportion of Fusobacterium-positive SSPs increased when metastasized from the distal to the proximal colon. Park^89^ and Ito's study^88^ found a stronger correlation between Fusobacterium spp. and CRC compared to less advanced lesions, with an increased presence of Fusobacterium spp. as histologic grading increased, which may be attributed to the new microenvironment of CRC tumors. SSPs typically exhibit mucus caps and overexpress mucin-forming proteins such as MUC6, MUC5aC, MUC17, and MUC2 genes associated with enhanced tumor metastasis^90^.

#### 4.2.2 Impact of Metabolites on the CRC Tumor Environment

##### 4.2.2.1 Bile Acids

**Figure 4:**
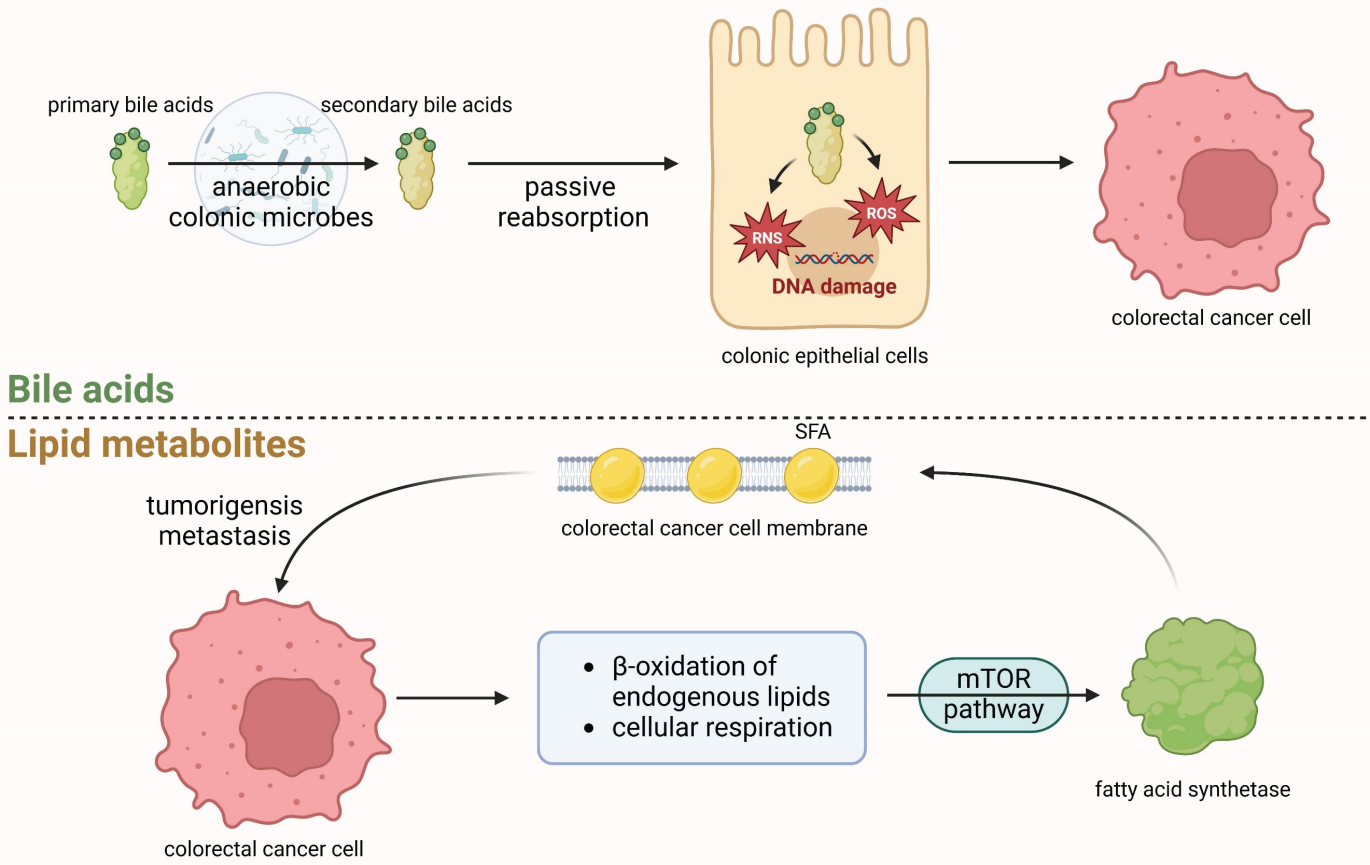
** Relevance of metabolites in the initiation and progression of colorectal cancer** Primary bile acids can undergo enzymatic cleavage by gut microbiota, particularly anaerobic colonic microbes, to form secondary bile acids. These secondary bile acids are subsequently passively reabsorbed into epithelial cells where they can induce an increase in reactive oxygen species (ROS) and reactive nitrogen species (RNS), leading to DNA damage in colonic epithelial cells and promoting the initiation of CRC. CRC cells respond by upregulating endogenous lipogenesis and cholesterol synthesis to support their proliferation, including activation of β-oxidation of endogenous lipids and cellular respiration^91^. These metabolic alterations activate fatty acid synthetase (FASN) through the mTOR pathway, enabling saturated fatty acids (SFA) incorporation into the cancer cell phospholipid membrane, thereby reducing susceptibility to free radicals and therapeutic drugs. This tumorigenic process may facilitate tumorigenesis and metastasis.

The levels of bile acids (BAs) and their metabolites in the intestinal lumen exhibit variations based on colonic location and are regulated by microbial enzymatic reactions. BAs and their metabolites can induce tumorigenesis^92^ (as shown in Fig. 4).

A nested case-control study in the EPIC cohort analyzed 17 types of BAs and their metabolites, finding a positive correlation between CRC risk and seven bound bile acid metabolites in plasma. This suggests that high levels of BAs can induce colon cancer. 93.

Importantly, previous studies have demonstrated a ten-fold higher concentration of primary bile acid-conjugated bile acids in the proximal colon compared to the distal colon, along with greater enzyme activity for DCA formation observed in cecal aspirate samples compared to rectal fecal samples^94^.

Subsequent investigations have further confirmed these findings by observing higher concentrations of BAs in the intestinal lumen of proximal colon cancer compared to distal colon cancer, as well as a greater diversity of BA species. The high ratio of glycineursodeoxycholic acid (GUDCA) to ursodeoxycholic acid (UDCA) was significantly associated with a worse 5-year OS, providing novel insights into the study of CRC-related microbial environment, which offers new avenues for CRC-related microbial research^95^.

Furthermore, recent clinical studies have also demonstrated that patients who undergo cholecystectomy are more susceptible to proximal colon cancer^96^.

The prolonged exposure of the proximal colon to bile acids and their metabolites occurs after cholecystectomy, as bile is no longer stored in its original site and its excretion does not follow the original feeding-related rhythm. This results in relatively less exposure of the distal colon and rectum.

##### 4.2.2.2 Lipid Metabolites

Lipids constitute a significant component of the human body's essential nutrients, and their metabolism is associated with the occurrence and progression of tumors through various metabolites. It is widely acknowledged that obesity, particularly in the EoCRC population^97^, is a risk factor for CRC^98^. This phenomenon is related to various lipid metabolites, such as saturated fatty acid (SFA), which is found in higher concentrations among obese individuals or MetS population. Such an elevation in SFA levels can induce oxidative stress, heightened lipotoxicity, and hypertriglyceridemia, among other effects. In order to meet the growing needs for continued proliferation, cancer cells can increase the uptake of exogenous lipids or upregulate endogenous lipogenesis and cholesterol synthesis^99^. As shown in Fig.4, tumor cells can promote tumorigenesis^100^,^101^ and even metastasis [102]through SFA^103^.

In addition to obesity, MetS manifests through dyslipidemia, abnormal blood glucose levels, and hypertension. Lipid metabolism abnormalities are closely linked to MetS and may contribute to the development and progression of proximal colon cancer^22^. Elevated SFA levels and other lipid metabolism disorders associated with MetS create a conducive environment for cancer cell growth and metastasis.

In order to investigate the role of lipids and their metabolites in tumor biological behavior, lipidomics-related research^104^,^105^ has recently emerged as a prominent area of focus in CRC research. Studies exploring lipid metabolites have also yielded unique findings in proximal colon cancer. Seyyedsalehi *et al.* collected dietary questionnaires from 865 CRC patients and 3206 healthy controls in the IROPICAN study. They discovered that there was an elevated association between high intake of industrial trans fatty acids and colon cancer risk after the age of 50, with the greatest increase observed in proximal colon cancer cases^106^. Another study analyzed tumor specimens from 246 CRC patients and identified fatty acid binding protein 4 (FABP4) expression in 37.0% of CRCs. FABP4 expression was significantly associated with age, proximal colon cancer subtype, nerve invasion, advanced pathological T stage, lymph node metastasis, advanced pathological TNM stage, as well as worse OS and DFS^107^.

Interestingly, the association between certain lipid metabolites and CRC appears to be gender-specific. One study^108^ investigating BAs and short-chain fatty acids (SCFAs) in serum samples revealed that women in the highest quartile of total SCFAs exhibited a 45% lower risk of CRC compared to those in the lowest quartile. Notably, this inverse correlation between total SCFAs and the risk of developing CRC was most pronounced among women with proximal colon cancer, potentially attributed to a gradual decline in SCFA concentration from the ascending to descending colon^109^,^110^.

Except for the body's endogenous lipid metabolism, there exists an additional crucial pathway involved in lipid metabolism, namely the intestinal microbiota. The aforementioned SCFAs serve as the primary metabolites generated by gut microbes through fermentation of insoluble dietary fiber, which can directly activate G-protein-coupled receptors (GPCRs), inhibit histone deacetylases^111^ and act as an energetic substrate linking dietary patterns and the intestinal microbiota to enhance intestinal health. In a comprehensive review investigating the impact of gut flora on CRC via SCFAs, Hou *et al.*^112^ summarized studies pertaining to this influence and concluded that both SCFAs and SCFA-producing bacteria exhibit significantly reduced abundance in CRC cases; moreover, supplementation with SCFA-producing probiotics has been shown to impede intestinal tumor development. Furthermore, modulation utilizing SCFAs as a guide in mouse and human CRC models enhances their response to chemotherapy and immunotherapy^113^,^114^. The intricate relationship between SCFAs and CRC may provide novel insights into diagnosing, treating, and preventing this disease.

##### 4.2.2.3 Amino Acids

Amino acids are abundant circulating metabolites and constitute proteins, serve as precursors for signaling molecules, and act as an important energy source through the citric acid cycle. Certain amino acids may also contribute to cancer progression^115^ while significant alterations in blood amino acid concentrations have been widely observed in patients with CRC^116^. For example, levels of amino acids such as glutamine, citrulline, alanine, and histidine inversely correlate with the stage of disease progression^117^,^118^. Moreover, valine and leucine have been identified as among the metabolites that can distinguish CRC cases^119^.

In a comprehensive study^120^, data from two large prospective cohorts, EPIC and UK Biobank (UKB), were combined. Notably, higher levels of circulating histidine demonstrated an association with reduced risk of CRC while glutamine exhibited a borderline inverse relationship. These findings suggest the need for further investigation into the potential involvement of histidine metabolism and glutamine metabolism in the pathogenesis mechanism of CRC.

However, consensus regarding variations in amino acid profiles across different anatomical locations in CRC has not been reached yet. Only one study statistically indicated differences in amino acid composition between proximal colon cancer and distal colon cancer^121^; however, additional relevant research is required to validate this conclusion.

## 5. Management

The current treatments for proximal colon cancer primarily encompass surgical and pharmacological interventions.

### 5.1 Surgical Treatment

Radical surgery is predominantly employed for colon cancer-related proximal colon cancer, with the extent of resection varying based on tumor location. Minimally invasive surgery has witnessed significant advancements and laparoscopic procedures currently represent the preferred surgical approach for proximal colon cancer treatment. The most controversial point in surgery currently focuses on D3- lymphadenectomy dissection.

The controversy surrounding whether proximal colon cancer patients should undergo D3-lymphadenectomy persists due to the higher proportion of tumors invading central lymph nodes in these patients compared to distal colon cancer patients, as well as the increased occurrence of tumor cells metastasizing through lymph nodes. 122. However, relevant studies have found that for stage III proximal colon cancer patients, the number of positive lymph nodes holds stronger predictive prognostic value than their distribution^123^. Therefore, high-quality research evidence is still required to determine whether radical D3 lymph node resection should be performed on proximal colon cancer patients. Except for the decision on performing D3-lymphadenectomy, there are also controversies regarding the boundaries of intraoperative D3-lymphadenectomy. Currently, three main perspectives exist on these boundaries: left side of the superior mesenteric vein (SMV), middle of the superior mesenteric artery (SMA), and left side of SMA. The key point under debate is whether it is possible to achieve a clean lymph node dissection at the third station while minimizing intraoperative bleeding and damage to autonomic nerves around SMA in order to shorten postoperative recovery time for gastrointestinal function^124^-^126^.

### 5.2 Pharmacological Treatments

The drug treatment of CRC can be divided into three main categories: chemotherapy drugs, immunotherapy drugs, and molecular targeted agents. In terms of indications for drug treatment in colon cancer, it is recommended for stage II patients with high-risk factors and those with stage III or more advanced stages. The first- and second-line options for drug treatment involve chemotherapy regimens based on fluorouracil, oxaliplatin, and irinotecan, either alone or in combination with macromolecular targeted drugs. As a third-line standard treatment approach, a fruquintinib-based single-agent regimen is typically employed. Based on the differences in biological characteristics between proximal colon cancer and distal colon cancer, since 2020, the NCCN guidelines have clearly proposed distinct drug treatment strategies for colon cancer based on anatomical location^127^.

#### 5.2.1 Chemotherapy

The proximal colon cancer exhibits heightened sensitivity to 5-fluorouracil chemotherapy regimens due to the prevalent occurrence of sporadic MSI-positive tumors, which are often accompanied by hypermethylation of the hMLH1 gene and positive for CIMP tumors characterized by concurrent methylation of multiple CpG islands. Notably, proximal colon cancer demonstrates a significantly higher DNA hypermethylation rate (4 to 13 times) compared to distal colon cancer. Among patients with locally advanced disease, those with MSI-positive tumors derive greater benefits from 5-fluorouracil-based chemotherapy. Consequently, proximal colon cancer patients benefit more from 5-fluorouracil-based chemotherapy than distal colon cancer patients^128^. On the contrary, the proportion of p53 overexpression in proximal colon cancer patients is lower than that in distal colon cancer, and the chemotherapy effect of oxaliplatin is better in CRC with p53 overexpression, so proximal colon cancer patients benefit less from oxaliplatin-based chemotherapy regimens^129^. Based on these findings, it can be inferred that the FOLFIRI (irinotecan + leucovorin + 5-fluorouracil) adjuvant chemotherapy regimen confers greater advantages for proximal colon cancer treatment than distal colon cancer cases, while the opposite holds true for the FOLFOX (5-fluorouracil + leucovorin + oxaliplatin) regimen.

#### 5.2.2 Immunotherapy Agents

Current immunotherapeutic agents for CRC primarily target two mechanisms, namely programmed death receptor-1 (PD-1) and cytotoxic T lymphocyte-associated antigen-4 (CTLA-4). The PD-1 monoclonal antibody pembrolizumab has demonstrated superior efficacy in mCRC patients with dMMR MSI-H, with significantly prolonged progression-free survival compared to chemotherapy while also presenting fewer treatment-related adverse events^130^. However, considering the relatively high prevalence of proficient mismatch repair proteins (pMMR) within the entire CRC population, a substantial proportion of patients do not derive benefits from immunotherapy. Therefore, further investigation into novel targeted therapies holds great promise.

#### 5.2.3 Molecularly Targeted Agents

CRC molecular targeted drug therapy is widely used, but the effect of targeted therapy varies depending on the primary location of CRC. At present, the commonly used targeted drugs in clinical practice are mainly anti-vascular endothelial growth factor (VEGF) inhibitors and anti-EGFR. The representative drugs are bevacizumab and cetuximab. In patients with proximal colon cancer, the combination of chemotherapeutic agents and anti-VEGF or anti-EGFR monoclonal antibodies is less effective than in distal colon cancer^131^, but still superior to regimens consisting solely of chemotherapeutic agents^132^. This combination therapy has the potential to improve patients' progression-free survival (PFS) and OS^133^.

As aforementioned, BRAF is more common in proximal colon cancer than distal colon cancer, and most mutations are BRAF^V600E^. A clinical trial demonstrated that colorectal cancer with BRAF^V600E^ gene mutations responded to the combination treatment of vemurafenib with cetuximab and irinotecan^134^. Patients used vemurafenib + cetuximab + irinotecan (VAF group) has better PFS than cetuximab + irinotecan (control group). They also tested circulating tumor DNA (ctDNA) before and after treatment. 87% of VAF group patients demonstrated a reduction of BRAF^V600E^ in the experimental arm, whereas no patients in the control group demonstrated reduction in ctDNA levels (P <0.001). Moreover, in this clinical trial, the proportion of proximal colon cancer higher than distal colon cancer and rectal cancer, but they didn't perform subgroup analyses according to tumor location.

KRAS is another common mutations gene in proximal colon cancer, which also related with metastasis of colorectal cancer^135^. Therefore, KRAS targeted agents worth more attention among proximal colon cancer. Recently, targeted therapies for KRAS mutations are focused on the KRAS^G12C^ mutation, with representative drugs such as sotorasib and adagrasib. These drugs were initially used in non-small cell lung cancer, with less favorable outcomes observed in colorectal cancer as compared to non-small cell lung cancer. Interestingly, further exploration has revealed that resistance to KRAS^G12C^ inhibitors stems from EGFR signaling, which is an upstream signal of KRAS. Consequently, it has been proposed that simultaneous inhibition of EGFR and KRAS^G12C^ should be pursued to overcome resistance to KRAS^G12C^ inhibitors in colorectal tumors^136^. Corresponding clinical trials have been conducted in a population of chemotherapy-refractory metastatic colorectal cancer patients, demonstrating that the combination of cetuximab (anti-EGFR monoclonal antibody) and KRAS^G12C^ inhibitor adagrasib is more effective than adagrasib monotherapy^137^. Additionally, another clinical trial demonstrated that increasing the dosage of the KRAS^G12C^ inhibitor sotorasib within a safe range can improve patients' PFS^138^. This trial also explored that proximal colon cancer subgroup treated with 960mg sorotasib + panitumumab has less disease-progression or death, comparing with distal colon cancer treated with same scheme. Therefore, it is valuable to select different molecular targeted therapies for treatment based on the location of the tumor. Currently, not every clinical trial focuses on the therapeutic effects in patients with tumors at different locations, and this is also one of the future directions for clinical trials or research.

#### 5.2.4 Alternative Therapies

Although the drug treatment of CRC has reached a relatively advanced stage, it still faces challenges such as distant metastasis and tumor drug resistance, which are common in most malignant tumors. Cutting-edge research methods such as multi-omics have provided new directions for drug treatment of CRC, especially for mCRC. Relevant studies have compared the disparities between genomics and proteomics in CRC and made novel findings at the phosphorylated protein and protein levels. For instance, one study^139^ utilized phosphoproteomic data analysis to propose a theoretical foundation for targeting retinoblastoma protein (Rb) phosphorylation in colon cancer; this study also observed reduced CD8 T cell infiltration in MSI-H tumors along with increased glycolysis, suggesting that glycolysis could be a potential target to overcome immune checkpoint blockade resistance in MSI-H tumors. Another study^140^ involving 480 clinical samples from 146 Chinese CRC patients, including 70 mCRC patients, found high genetic similarity between metastatic tissue and primary tumor but not at the proteomic level; kinase network analysis revealed significant heterogeneity between primary CRCs and their liver metastases, providing insights into predicting drug response to some extent. Further research on more personalized treatment regimens for CRC, including for proximal colon cancer, is still needed with the aim of improving treatment outcomes^141^.

## 6. Conclusion

With the improvement of people's living standards and advancements in medical care, CRC accounts for an increasing proportion of common malignant tumors and has garnered growing attention. Proximal colon cancer, as a distinct anatomical subtypes with a poorer prognosis, exhibits unique epidemiological characteristics, clinicopathological manifestations, biological behavioral features, and therapeutic responses. In the current era of rapid scientific research techniques and concepts, there remains ample room for further investigation into proximal colon cancer.

Through deeper research, clinicians should gradually recognize that proximal colon cancer not only signifies differences in anatomical location, but also encompasses a series of disparate biological behaviors compared to distal colon cancer and RC. A clearer understanding can greatly contribute to the treatment, diagnosis, and even prevention of proximal colon cancer.

## Figures and Tables

**Figure 1:**
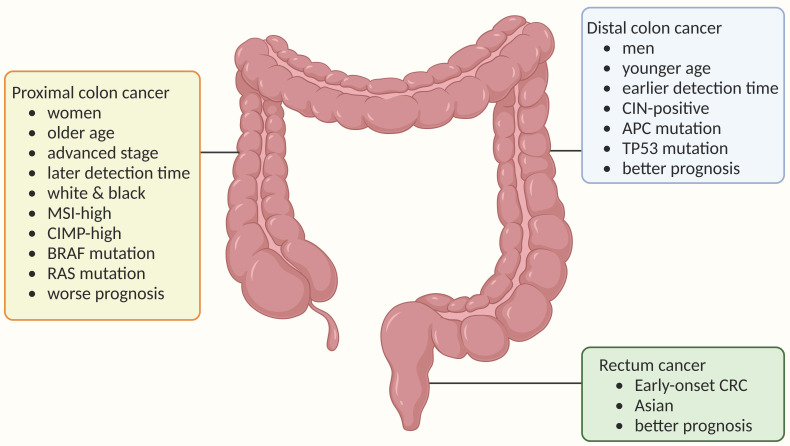
** Anatomical subtypes of colorectal cancer and their associations with epidemiological features, clinical features and tumor molecular features.** The colorectum is anatomically divided into three segments: the proximal colon, which extends from the caecum through the ascending colon to the transverse colon; the distal colon, consisting of the descending colon and sigmoid colon; and the rectum. Colorectal cancer (CRC) exhibits etiological heterogeneity based on tumor location. Demographically, proximal colon cancer is more prevalent in women, older individuals, and white and black populations; distal colon cancer occurs more frequently in men and younger individuals; while rectal cancer is predominant among early-onset cases (diagnosed before age 50 years) and Asian populations. In terms of tumor molecular markers, proximal colon cancer shows enrichment for subtypes characterized by microsatellite instability-high status (MSI-H), CpG island methylator phenotype-high status (CIMP-H), BRAF mutation, or RAS mutation; whereas distal colon cancer is associated with chromosomal instability (CIN)-positive subtype. Understanding the epidemiological trends of proximal colon cancer helps contextualize its distinct clinical presentations.

**Figure 2:**
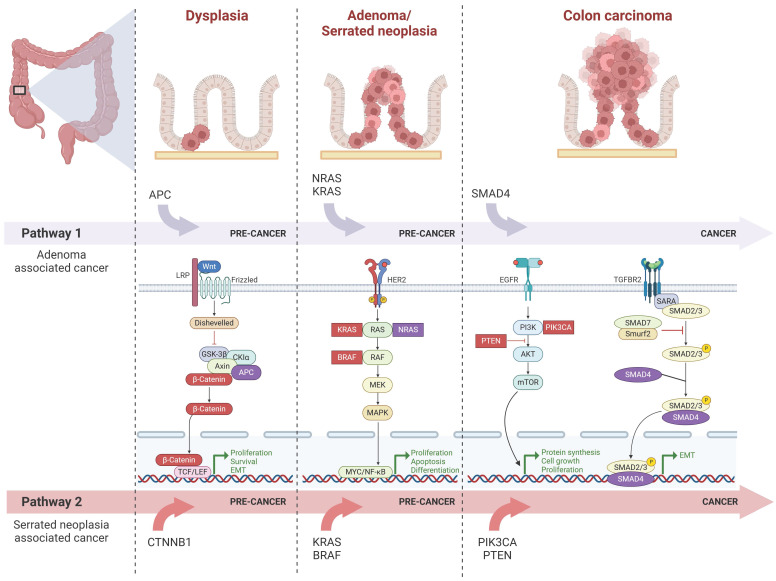
**Canonical molecular pathways underlying the initiation and progression of proximal colon cancer** Proximal colon cancer typically arises from adenoma or serrated neoplasia. As depicted in the figure, pathway 1 illustrates the commonly mutated genes and molecular pathways involved in the progression from adenoma to carcinoma, while pathway 2 represents those associated with the progression from serrated neoplasia to cancer. Notably, during the dysplasia stage, the Wnt signaling pathway predominantly operates as a key molecular pathway; APC exhibits closer association with carcinogenesis mechanisms linked to adenoma, whereas β-catenin is more closely related to those associated with serrated neoplasia. In both adenoma and serrated neoplasia stages, HER-2 signaling pathway plays significant role. Mutations in NRAS and KRAS are particularly relevant to adenoma-associated carcinogenesis, whereas KRAS and BRAF mutations are more closely tied to serrated neoplasia. During cancer progression, major molecular pathways include EGFR signaling and TGF-β signaling pathways. Among these, SMAD4 demonstrates stronger correlation with adenoma's carcinogenesis mechanism while PIK3CA and PTEN exhibit closer associations with serrated neoplasia's carcinogenesis mechanism.

**Table 1:** Characteristics of common CRC molecular markers in proximal colon cancer

	Related Pathway	Features in CRC	Features in proximal colon cancer
MMR	DNA mismatch repair protein	About 15% of the CRC is MSI^142^, patients with MSI have better prognosis.	MSI is much more prevalent in proximal colon cancer, with the highest proportion in patients with stage II proximal colon cancer, up to 20% to 25%^143^. MSI-H patients have prolonged survival time.
RAS	RAS-RAF-MEK-ARK-MAPK signaling pathway^144^	There are three types of mutations, KRAS, NRAS, and HRAS; when mutated, they can activate the downstream of the EGFR signaling pathway from the alternative pathway and promote tumorigenesis.	KRAS mutations are more common in proximal colon cancer; anti-EGFR monoclonal antibody therapy is less effective in patients with mutated RAS.
BRAF	RAS-RAF-MEK-ARK-MAPK signaling pathway^144^	Same as above, the most common form of mutation is V600E, which activates the above pathways after mutation and promotes tumorigenesis.	More common in proximal colon cancer; higher proportion of MSI-H in patients with BRAF mutations.
PIK3CA	AKT pathway	Oncogene, activated by mutation.	Higher frequency in proximal colon cancer, CIMP-L and KRAS mutations.
SMAD4	TGF-β pathway	Tumor suppressor, loss of oncogenic function by mutation, leading to carcinogenesis.	More common in proximal colon cancer; More common in colorectal adenocarcinomas with a mucinous component.
CTNNB1	Wnt signaling pathway	Abnormal activation after mutation, leading to cancer.	More common in proximal colon cancer; The splenic flexure of the colon is the most common location.
PTEN	AKT pathway	Tumor suppressor genes, lose their antagonistic effect on tyrosine kinase phosphorylation when mutated or deleted, then lead to the carcinogenesis.	Most common in proximal colon cancer; more common in mucus histologically; associated with MSI-H, CIMP-H and BRAF mutations.

## Abbreviations

AC: classic colorectal adenocarcinoma
AWMC: adenocarcinoma with mucinous component
BAs: bile acids
CIMP: CpG island methylator phenotype
CIMP-H: CpG island methylator phenotype-high status
CIN: chromosomal instability
CMS: consensus molecular subtype
CRC: colorectal cancer
CSS: cancer-specific survival
ctDNA: circulating tumor DNA
CTLA-4: cytotoxic T lymphocyte-associated antigen-4
DFS: disease-free survival
EGFR: epidermal growth factor receptor
FABP4: fatty acid binding protein 4
FASN: fatty acid synthetase
GPCRs: G-protein-coupled receptors
GUDCA: glycineursodeoxycholic acid
HGF: hepatocyte growth factor
HNPCC: hereditary non-polyposis colorectal cancer
IL-6: interleukin-6
mCRC: metastatic colorectal cancer
MetS: metabolic syndrome
MGMT: O-6-methylguanine-DNA methyltransferase
MSI: microsatellite instability
MSI-H: microsatellite instability-high
MSS: microsatellite-stable
OS: overall survival
PCWBR2: putative cell wall binding repeat 2
PD-1: programmed death receptor-1
PFS: progression-free survival
PI3Ks: class I phosphatidylinositol-3-kinases
pMMR: proficient mismatch repair proteins
PTEN: phosphatase and tensin homologue deleted on chromosome ten
PTP: protein tyrosine phosphatases
Rb: retinoblastoma protein
RC: rectal cancer
RNS: reactive nitrogen species
ROS: reactive oxygen species
SCFAs: short-chain fatty acids
SFA: saturated fatty acids
SMA: superior mesenteric artery
SMV: superior mesenteric vein
SSP: sessile serrated polyps
TME: tumor micro-environment
TSA: traditional serrated adenomas
UDCA: ursodeoxycholic acid
UKB: UK Biobank
VEGF: vascular endothelial growth factor

## References

[1] Siegel RL, Wagle NS, Cercek A (2023). Colorectal cancer statistics, 2023. CA: A Cancer Journal for Clinicians.

[2] Qu R, Ma Y, Zhang Z (2022). Increasing burden of colorectal cancer in China. Lancet Gastroenterol Hepatol.

[3] Duraes LC, Steele SR, Valente MA (2022). Right colon, left colon, and rectal cancer have different oncologic and quality of life outcomes. Int J Colorectal Dis.

[4] Yang SY, Cho MS, Kim NK (2018). Difference between right-sided and left-sided colorectal cancers: from embryology to molecular subtype. Expert Review of Anticancer Therapy.

[5] Araki K, Furuya Y, Kobayashi M (1996). Comparison of mucosal microvasculature between the proximal and distal human colon. J Electron Microsc (Tokyo).

[6] Tricoli JV, Rall LB, Karakousis CP (1986). Enhanced levels of insulin-like growth factor messenger RNA in human colon carcinomas and liposarcomas. Cancer Res.

[7] Reichmann A, Levin B, Martin P (1982). Human large-bowel cancer: correlation of clinical and histopathological features with banded chromosomes. Int J Cancer.

[8] Qiu MZ, Pan WT, Lin JZ (2018). Comparison of survival between right-sided and left-sided colon cancer in different situations. Cancer Med.

[9] Yang J, Du XL, Li ST (2016). Characteristics of Differently Located Colorectal Cancers Support Proximal and Distal Classification: A Population-Based Study of 57,847 Patients. PLoS One.

[10] Luo C, Cen S, Ding G (2019). Mucinous colorectal adenocarcinoma: clinical pathology and treatment options. Cancer Commun (Lond).

[11] Zhu L, Ling C, Xu T (2021). Clinicopathological Features and Survival of Signet-Ring Cell Carcinoma and Mucinous Adenocarcinoma of Right Colon, Left Colon, and Rectum. Pathol Oncol Res.

[12] Loree JM, Pereira AAL, Lam M (2018). Classifying Colorectal Cancer by Tumor Location Rather than Sidedness Highlights a Continuum in Mutation Profiles and Consensus Molecular Subtypes. Clinical Cancer Research.

[13] Guinney J, Dienstmann R, Wang X (2015). The consensus molecular subtypes of colorectal cancer. Nat Med.

[14] Lee MS, Menter DG, Kopetz S (2017). Right Versus Left Colon Cancer Biology: Integrating the Consensus Molecular Subtypes. J Natl Compr Canc Netw.

[15] Benson AB, Venook AP, Al-Hawary MM (2021). Colon Cancer, Version 2.2021, NCCN Clinical Practice Guidelines in Oncology. J Natl Compr Canc Netw.

[16] Murphy N, Ward HA, Jenab M (2019). Heterogeneity of Colorectal Cancer Risk Factors by Anatomical Subsite in 10 European Countries: A Multinational Cohort Study. Clinical Gastroenterology and Hepatology.

[17] Shi JF, Wang L, Ran JC (2021). Clinical characteristics, medical service utilization, and expenditure for colorectal cancer in China, 2005 to 2014: Overall design and results from a multicenter retrospective epidemiologic survey. Cancer.

[18] Qu R, Ma Y, Tao L (2021). Features of colorectal cancer in China stratified by anatomic sites: A hospital-based study conducted in university-affiliated hospitals from 2014 to 2018. Chin J Cancer Res.

[19] Nakagawa H, Ito H, Hosono S (2017). Changes in trends in colorectal cancer incidence rate by anatomic site between 1978 and 2004 in Japan. Eur J Cancer Prev.

[20] Shin A, Kim KZ, Jung KW (2012). Increasing trend of colorectal cancer incidence in Korea, 1999-2009. Cancer Res Treat.

[21] Lu Y, Ness-Jensen E, Hveem K (2015). Metabolic predispositions and increased risk of colorectal adenocarcinoma by anatomical location: a large population-based cohort study in Norway. Am J Epidemiol.

[22] Chiu HM, Lin JT, Shun CT (2007). Association of metabolic syndrome with proximal and synchronous colorectal neoplasm. Clin Gastroenterol Hepatol.

[23] Hirode G, Wong RJ (2020). Trends in the Prevalence of Metabolic Syndrome in the United States, 2011-2016. JAMA.

[24] Scuteri A, Laurent S, Cucca F (2015). Metabolic syndrome across Europe: different clusters of risk factors. Eur J Prev Cardiol.

[25] Saklayen MG (2018). The Global Epidemic of the Metabolic Syndrome. Curr Hypertens Rep.

[26] Cheng L, Eng C, Nieman LZ (2011). Trends in colorectal cancer incidence by anatomic site and disease stage in the United States from 1976 to 2005. Am J Clin Oncol.

[27] Dekker E, Rex DK (2018). Advances in CRC Prevention: Screening and Surveillance. Gastroenterology.

[28] Cucino C, Buchner AM, Sonnenberg A (2002). Continued Rightward Shift of Colorectal Cancer. Diseases of the Colon & Rectum.

[29] Thompson MR, O'Leary DP, Flashman K (2017). Clinical assessment to determine the risk of bowel cancer using Symptoms, Age, Mass and Iron deficiency anaemia (SAMI). Br J Surg.

[30] Cai X, Gu D, Chen M (2018). The effect of the primary tumor location on the survival of colorectal cancer patients after radical surgery. Int J Med Sci.

[31] Weiss JM, Pfau PR, O'Connor ES (2011). Mortality by stage for right- versus left-sided colon cancer: analysis of surveillance, epidemiology, and end results-Medicare data. J Clin Oncol.

[32] Jernvall P, Mäkinen MJ, Karttunen TJ (1999). Microsatellite instability: impact on cancer progression in proximal and distal colorectal cancers. Eur J Cancer.

[33] Yahagi M, Okabayashi K, Hasegawa H (2016). The Worse Prognosis of Right-Sided Compared with Left-Sided Colon Cancers: a Systematic Review and Meta-analysis. J Gastrointest Surg.

[34] Murphy G, Devesa SS, Cross AJ (2011). Sex disparities in colorectal cancer incidence by anatomic subsite, race and age. Int J Cancer.

[35] Leandro Fórnias Machado de Rezende, Sá TH de, Markozannes G (2018). Physical activity and cancer: an umbrella review of the literature including 22 major anatomical sites and 770 000 cancer cases. Br J Sports Med.

[36] Mehta RS, Song M, Nishihara R (2017). Dietary Patterns and Risk of Colorectal Cancer: Analysis by Tumor Location and Molecular Subtypes. Gastroenterology.

[37] Bai X, Wei H, Liu W (2022). Cigarette smoke promotes colorectal cancer through modulation of gut microbiota and related metabolites. Gut.

[38] Nitsche U, Zimmermann A, Späth C (2013). Mucinous and signet-ring cell colorectal cancers differ from classical adenocarcinomas in tumor biology and prognosis. Ann Surg.

[39] Consorti F, Lorenzotti A, Midiri G (2000). Prognostic significance of mucinous carcinoma of colon and rectum: a prospective case-control study. J Surg Oncol.

[40] Benedix F, Meyer F, Kube R (2010). [Right- and left-sided colonic cancer - different tumour entities]. Zentralbl Chir.

[41] Luo XJ, Zhao Q, Liu J (2021). Novel Genetic and Epigenetic Biomarkers of Prognostic and Predictive Significance in Stage II/III Colorectal Cancer. Mol Ther.

[42] Crockett SD, Nagtegaal ID (2019). Terminology, Molecular Features, Epidemiology, and Management of Serrated Colorectal Neoplasia. Gastroenterology.

[43] Cancer Genome Atlas Network (2012). Comprehensive molecular characterization of human colon and rectal cancer. Nature.

[44] Rosty C, Young JP, Walsh MD (2013). PIK3CA activating mutation in colorectal carcinoma: associations with molecular features and survival. PLoS One.

[45] Loree JM, Pereira AAL, Lam M (2018). Classifying Colorectal Cancer by Tumor Location Rather than Sidedness Highlights a Continuum in Mutation Profiles and Consensus Molecular Subtypes. Clin Cancer Res.

[46] Huyghe JR, Harrison TA, Bien SA (2021). Genetic architectures of proximal and distal colorectal cancer are partly distinct. Gut.

[47] Taieb J, Le Malicot K, Shi Q (2017). Prognostic Value of BRAF and KRAS Mutations in MSI and MSS Stage III Colon Cancer. J Natl Cancer Inst.

[48] Venderbosch S, Nagtegaal ID, Maughan TS (2014). Mismatch repair status and BRAF mutation status in metastatic colorectal cancer patients: a pooled analysis of the CAIRO, CAIRO2, COIN, and FOCUS studies. Clin Cancer Res.

[49] Fennell LJ, Jamieson S, McKeone D (2018). MLH1-93 G/a polymorphism is associated with MLH1 promoter methylation and protein loss in dysplastic sessile serrated adenomas with BRAFV600E mutation. BMC Cancer.

[50] Lu HY, Lin RT, Zhou GX (2017). Critical Role of p53 and K-ras in the Diagnosis of Early Colorectal Cancer: a One-year, Single-center Analysis. Int J Med Sci.

[51] Ryan MB, Corcoran RB (2018). Therapeutic strategies to target RAS-mutant cancers. Nat Rev Clin Oncol.

[52] Harada K, Hiraoka S, Kato J (2007). Genetic and epigenetic alterations of Ras signalling pathway in colorectal neoplasia: analysis based on tumour clinicopathological features. Br J Cancer.

[53] Negri F, Bottarelli L, de'Angelis GL (2022). KRAS: A Druggable Target in Colon Cancer Patients. International Journal of Molecular Sciences.

[54] Taieb J, Balogoun R, Le Malicot K (2017). Adjuvant FOLFOX +/- cetuximab in full RAS and BRAF wildtype stage III colon cancer patients. Ann Oncol.

[55] Roviello G, D'Angelo A, Petrioli R (2020). Encorafenib, Binimetinib, and Cetuximab in BRAF V600E-Mutated Colorectal Cancer. Translational Oncology.

[56] Taieb J, Zaanan A, Le Malicot K (2016). Prognostic Effect of BRAF and KRAS Mutations in Patients With Stage III Colon Cancer Treated With Leucovorin, Fluorouracil, and Oxaliplatin With or Without Cetuximab: A Post Hoc Analysis of the PETACC-8 Trial. JAMA Oncol.

[57] Siena S, Sartore-Bianchi A, Di Nicolantonio F (2009). Biomarkers predicting clinical outcome of epidermal growth factor receptor-targeted therapy in metastatic colorectal cancer. J Natl Cancer Inst.

[58] Schirripa M, Cohen SA, Battaglin F (2018). Biomarker-driven and molecular targeted therapies for colorectal cancers. Semin Oncol.

[59] Sveen A, Kopetz S, Lothe RA (2020). Biomarker-guided therapy for colorectal cancer: strength in complexity. Nat Rev Clin Oncol.

[60] Del Rio P, Rossini M, Giuffrida M (2020). Rightward shift in colorectal cancer: experience in 1101 patients. Minerva Chir.

[61] Madsen RR, Vanhaesebroeck B, Semple RK (2018). Cancer-Associated PIK3CA Mutations in Overgrowth Disorders. Trends Mol Med.

[62] Day FL, Jorissen RN, Lipton L (2013). PIK3CA and PTEN gene and exon mutation-specific clinicopathologic and molecular associations in colorectal cancer. Clin Cancer Res.

[63] Yaeger R, Chatila WK, Lipsyc MD (2018). Clinical Sequencing Defines the Genomic Landscape of Metastatic Colorectal Cancer. Cancer Cell.

[64] Chen J, Zhou L, Gao J (2020). Clinicopathological Characteristics and Mutation Spectrum of Colorectal Adenocarcinoma With Mucinous Component in a Chinese Cohort: Comparison With Classical Adenocarcinoma. Front Oncol.

[65] Albuquerque C, Baltazar C, Filipe B (2010). Colorectal cancers show distinct mutation spectra in members of the canonical WNT signaling pathway according to their anatomical location and type of genetic instability. Genes Chromosomes Cancer.

[66] Naguib A, Cooke JC, Happerfield L (2011). Alterations in PTEN and PIK3CA in colorectal cancers in the EPIC Norfolk study: associations with clinicopathological and dietary factors. BMC Cancer.

[67] Dekker E, Tanis PJ, Vleugels JLA (2019). Colorectal cancer. The Lancet.

[68] Jin K, Ren C, Liu Y (2020). An update on colorectal cancer microenvironment, epigenetic and immunotherapy. International Immunopharmacology.

[69] Thursby E, Juge N (2017). Introduction to the human gut microbiota. Biochem J.

[70] Tremaroli V, Bäckhed F (2012). Functional interactions between the gut microbiota and host metabolism. Nature.

[71] Helmink BA, Khan MAW, Hermann A (2019). The microbiome, cancer, and cancer therapy. Nat Med.

[72] Park EM, Chelvanambi M, Bhutiani N (2022). Targeting the gut and tumor microbiota in cancer. Nat Med.

[73] Kim J, Lee HK (2021). Potential Role of the Gut Microbiome In Colorectal Cancer Progression. Front Immunol.

[74] Qu R, Zhang Y, Ma Y (2023). Role of the Gut Microbiota and Its Metabolites in Tumorigenesis or Development of Colorectal Cancer. Adv Sci (Weinh).

[75] Martin OCB, Bergonzini A, D'Amico F (2019). Infection with genotoxin-producing Salmonella enterica synergises with loss of the tumour suppressor APC in promoting genomic instability via the PI3K pathway in colonic epithelial cells. Cell Microbiol.

[76] Mima K, Nishihara R, Qian ZR (2016). Fusobacterium nucleatum in colorectal carcinoma tissue and patient prognosis. Gut.

[77] Wei Z, Cao S, Liu S (2016). Could gut microbiota serve as prognostic biomarker associated with colorectal cancer patients' survival?. A pilot study on relevant mechanism. Oncotarget.

[78] Deng Z, Mu J, Tseng M (2015). Enterobacteria-secreted particles induce production of exosome-like S1P-containing particles by intestinal epithelium to drive Th17-mediated tumorigenesis. Nat Commun.

[79] Long X, Wong CC, Tong L (2019). Peptostreptococcus anaerobius promotes colorectal carcinogenesis and modulates tumour immunity. Nat Microbiol.

[80] Cougnoux A, Dalmasso G, Martinez R (2014). Bacterial genotoxin colibactin promotes colon tumour growth by inducing a senescence-associated secretory phenotype. Gut.

[81] Gao R, Kong C, Huang L (2017). Mucosa-associated microbiota signature in colorectal cancer. Eur J Clin Microbiol Infect Dis.

[82] Flemer B, Lynch DB, Brown JMR (2017). Tumour-associated and non-tumour-associated microbiota in colorectal cancer. Gut.

[83] Dejea CM, Wick EC, Hechenbleikner EM (2014). Microbiota organization is a distinct feature of proximal colorectal cancers. Proc Natl Acad Sci U S A.

[84] Johnson CH, Dejea CM, Edler D (2015). Metabolism links bacterial biofilms and colon carcinogenesis. Cell Metab.

[85] DeDecker L, Coppedge B, Avelar-Barragan J (2021). Microbiome distinctions between the CRC carcinogenic pathways. Gut Microbes.

[86] Yang JF, Tang SJ, Lash RH (2015). Anatomic distribution of sessile serrated adenoma/polyp with and without cytologic dysplasia. Arch Pathol Lab Med.

[87] Yu J, Chen Y, Fu X (2016). Invasive Fusobacterium nucleatum may play a role in the carcinogenesis of proximal colon cancer through the serrated neoplasia pathway. Int J Cancer.

[88] Ito M, Kanno S, Nosho K (2015). Association of Fusobacterium nucleatum with clinical and molecular features in colorectal serrated pathway. Int J Cancer.

[89] Park CH, Han DS, Oh YH (2016). Role of Fusobacteria in the serrated pathway of colorectal carcinogenesis. Sci Rep.

[90] Delker DA, McGettigan BM, Kanth P (2014). RNA sequencing of sessile serrated colon polyps identifies differentially expressed genes and immunohistochemical markers. PLoS One.

[91] Qu R, Zhang Z, Fu W (2024). Lifestyle-Associated Risk Factors and Gastrointestinal Cancers: Targeting Potential of the Gut Microbe-Host Crosstalk-Based Metabolic Processes. Gastroenterology.

[92] Venturi M, Hambly RJ, Glinghammar B (1997). Genotoxic activity in human faecal water and the role of bile acids: a study using the alkaline comet assay. Carcinogenesis.

[93] Kühn T, Stepien M, López-Nogueroles M (2020). Prediagnostic Plasma Bile Acid Levels and Colon Cancer Risk: A Prospective Study. J Natl Cancer Inst.

[94] Thomas LA, Veysey MJ, French G (2001). Bile acid metabolism by fresh human colonic contents: a comparison of caecal versus faecal samples. Gut.

[95] Cai Y, Shen X, Lu L (2022). Bile acid distributions, sex-specificity, and prognosis in colorectal cancer. Biol Sex Differ.

[96] Ábrahám S, Németh T, Benkő R (2020). Evaluating the distribution of the locations of colorectal cancer after appendectomy and cholecystectomy. World J Surg Oncol.

[97] Spaander MCW, Zauber AG, Syngal S (2023). Young-onset colorectal cancer. Nat Rev Dis Primers.

[98] Bardou M, Barkun AN, Martel M (2013). Obesity and colorectal cancer. Gut.

[99] Piccinin E, Cariello M, Moschetta A (2021). Lipid metabolism in colon cancer: Role of Liver X Receptor (LXR) and Stearoyl-CoA Desaturase 1 (SCD1). Mol Aspects Med.

[100] Chang L, Wu P, Senthilkumar R (2016). Loss of fatty acid synthase suppresses the malignant phenotype of colorectal cancer cells by down-regulating energy metabolism and mTOR signaling pathway. J Cancer Res Clin Oncol.

[101] Francipane MG, Lagasse E (2014). mTOR pathway in colorectal cancer: an update. Oncotarget.

[102] Gulhati P, Bowen KA, Liu J (2011). mTORC1 and mTORC2 regulate EMT, motility, and metastasis of colorectal cancer via RhoA and Rac1 signaling pathways. Cancer Res.

[103] Zaytseva YY, Harris JW, Mitov MI (2015). Increased expression of fatty acid synthase provides a survival advantage to colorectal cancer cells via upregulation of cellular respiration. Oncotarget.

[104] Ye C, Wang J, Tan S (2013). Meta-analysis of adiponectin polymorphisms and colorectal cancer risk. Int J Med Sci.

[105] Pakiet A, Kobiela J, Stepnowski P (2019). Changes in lipids composition and metabolism in colorectal cancer: a review. Lipids Health Dis.

[106] Seyyedsalehi MS, Collatuzzo G, Rashidian H (2022). Dietary Ruminant and Industrial Trans-Fatty Acids Intake and Colorectal Cancer Risk. Nutrients.

[107] Kim SH, Pyo JS, Son BK (2023). Clinicopathological significance and prognostic implication of nuclear fatty acid-binding protein 4 expression in colorectal cancer. Pathol Res Pract.

[108] Loftfield E, Falk RT, Sampson JN (2022). Prospective Associations of Circulating Bile Acids and Short-Chain Fatty Acids With Incident Colorectal Cancer. JNCI Cancer Spectr.

[109] Topping DL, Clifton PM (2001). Short-chain fatty acids and human colonic function: roles of resistant starch and nonstarch polysaccharides. Physiol Rev.

[110] Cummings JH, Pomare EW, Branch WJ (1987). Short chain fatty acids in human large intestine, portal, hepatic and venous blood. Gut.

[111] Bultman SJ (2014). Molecular pathways: gene-environment interactions regulating dietary fiber induction of proliferation and apoptosis via butyrate for cancer prevention. Clin Cancer Res.

[112] Hou H, Chen D, Zhang K (2022). Gut microbiota-derived short-chain fatty acids and colorectal cancer: Ready for clinical translation?. Cancer Lett.

[113] Ma X, Zhou Z, Zhang X (2020). Sodium butyrate modulates gut microbiota and immune response in colorectal cancer liver metastatic mice. Cell Biol Toxicol.

[114] He Y, Fu L, Li Y (2021). Gut microbial metabolites facilitate anticancer therapy efficacy by modulating cytotoxic CD8+ T cell immunity. Cell Metab.

[115] Lieu EL, Nguyen T, Rhyne S (2020). Amino acids in cancer. Exp Mol Med.

[116] Goveia J, Pircher A, Conradi LC (2016). Meta-analysis of clinical metabolic profiling studies in cancer: challenges and opportunities. EMBO Mol Med.

[117] Sirniö P, Väyrynen JP, Klintrup K (2019). Alterations in serum amino-acid profile in the progression of colorectal cancer: associations with systemic inflammation, tumour stage and patient survival. Br J Cancer.

[118] Geijsen AJMR, van Roekel EH, van Duijnhoven FJB (2020). Plasma metabolites associated with colorectal cancer stage: Findings from an international consortium. Int J Cancer.

[119] Geijsen AJMR, Brezina S, Keski-Rahkonen P (2019). Plasma metabolites associated with colorectal cancer: A discovery-replication strategy. Int J Cancer.

[120] Rothwell JA, Bešević J, Dimou N (2023). Circulating amino acid levels and colorectal cancer risk in the European Prospective Investigation into Cancer and Nutrition and UK Biobank cohorts. BMC Med.

[121] Feng Y, Gao Y, Yue CC (2022). Comparison between the left and right-sided colon cancer: A study based on clinical pathology and serum amino acid metabolism. Asian J Surg.

[122] Kataoka K, Beppu N, Shiozawa M (2020). Colorectal cancer treated by resection and extended lymphadenectomy: patterns of spread in left- and right-sided tumours. Br J Surg.

[123] Kim CH, Huh JW, Kim HR (2014). Prognostic comparison between number and distribution of lymph node metastases in patients with right-sided colon cancer. Ann Surg Oncol.

[124] Balciscueta Z, Balciscueta I, Uribe N (2021). D3-lymphadenectomy enhances oncological clearance in patients with right colon cancer. Results of a meta-analysis. Eur J Surg Oncol.

[125] Spasojevic M, Stimec BV, Dyrbekk APH (2013). Lymph node distribution in the d3 area of the right mesocolon: implications for an anatomically correct cancer resection. A postmortem study. Dis Colon Rectum.

[126] Nesgaard JM, Stimec BV, Soulie P (2018). Defining minimal clearances for adequate lymphatic resection relevant to right colectomy for cancer: a post-mortem study. Surg Endosc.

[127] Provenzale D, Ness RM, Llor X (2020). NCCN Guidelines Insights: Colorectal Cancer Screening, Version 2.2020. J Natl Compr Canc Netw.

[128] Tejpar S, Bosman F, Delorenzi M (2009). Microsatellite instability (MSI) in stage II and III colon cancer treated with 5FU-LV or 5FU-LV and irinotecan (PETACC 3-EORTC 40993-SAKK 60/00 trial). JCO.

[129] Sho S, Court CM, Winograd P (2017). A prognostic mutation panel for predicting cancer recurrence in stages II and III colorectal cancer. J Surg Oncol.

[130] André T, Shiu KK, Kim TW (2020). Pembrolizumab in Microsatellite-Instability-High Advanced Colorectal Cancer. N Engl J Med.

[131] Arnold D, Lueza B, Douillard JY (2017). Prognostic and predictive value of primary tumour side in patients with RAS wild-type metastatic colorectal cancer treated with chemotherapy and EGFR directed antibodies in six randomized trials. Ann Oncol.

[132] Wang ZX, Wu HX, He MM (2019). Chemotherapy With or Without Anti-EGFR Agents in Left- and Right-Sided Metastatic Colorectal Cancer: An Updated Meta-Analysis. J Natl Compr Canc Netw.

[133] You XH, Jiang YH, Fang Z (2020). Chemotherapy plus bevacizumab as an optimal first-line therapeutic treatment for patients with right-sided metastatic colon cancer: a meta-analysis of first-line clinical trials. ESMO Open.

[134] Kopetz S, Guthrie KA, Morris VK (2021). Randomized Trial of Irinotecan and Cetuximab With or Without Vemurafenib in BRAF-Mutant Metastatic Colorectal Cancer (SWOG S1406). J Clin Oncol.

[135] Nguyen B, Fong C, Luthra A (2022). Genomic characterization of metastatic patterns from prospective clinical sequencing of 25,000 patients. Cell.

[136] Amodio V, Yaeger R, Arcella P (2020). EGFR Blockade Reverts Resistance to KRASG12C Inhibition in Colorectal Cancer. Cancer Discov.

[137] Yaeger R, Weiss J, Pelster MS (2023). Adagrasib with or without Cetuximab in Colorectal Cancer with Mutated KRAS G12C. N Engl J Med.

[138] Fakih MG, Salvatore L, Esaki T (2023). Sotorasib plus Panitumumab in Refractory Colorectal Cancer with Mutated KRAS G12C. N Engl J Med.

[139] Vasaikar S, Huang C, Wang X (2019). Proteogenomic Analysis of Human Colon Cancer Reveals New Therapeutic Opportunities. Cell.

[140] Li C, Sun YD, Yu GY (2020). Integrated Omics of Metastatic Colorectal Cancer. Cancer Cell.

[141] Qu R, Zhang Z, Fu W (2024). Potential of Serum Glycoproteome Profiling in Prediction of Advanced Adenomas and Colorectal Carcinoma: Individual Heterogeneity Should Be Taken Into Account. Gastroenterology.

[142] Boland CR, Goel A (2010). Microsatellite instability in colorectal cancer. Gastroenterology.

[143] Klingbiel D, Saridaki Z, Roth AD (2015). Prognosis of stage II and III colon cancer treated with adjuvant 5-fluorouracil or FOLFIRI in relation to microsatellite status: results of the PETACC-3 trial. Ann Oncol.

[144] Li J, Ma X, Chakravarti D (2021). Genetic and biological hallmarks of colorectal cancer. Genes Dev.

